# Network pharmacology integrated with molecular docking and experimental validation elucidates the therapeutic potential of *Forsythiae Fructus* extract against hepatitis B virus-related hepatocellular carcinoma

**DOI:** 10.3389/fonc.2025.1571537

**Published:** 2025-06-24

**Authors:** Fuqing Chen, Yifan Cai, Changzhou Chen, Jianyin Zhou

**Affiliations:** ^1^ Department of Hepatobiliary Surgery, Xiamen Key Laboratory of Translational Medical of Digestive System Tumor, Fujian Provincial Key Laboratory of Chronic Liver Disease and Hepatocellular Carcinoma, Zhongshan Hospital of Xiamen University, School of Medicine, Xiamen University, Xiamen, Fujian, China; ^2^ Department of Gastrointestinal Surgery, Zhongshan Hospital of Xiamen University, School of Medicine, Xiamen University, Xiamen, Fujian, China; ^3^ Department Minimally Invasive and Interventional Oncology, Zhongshan Hospital of Xiamen University, School of Medicine, Xiamen University, Xiamen, Fujian, China

**Keywords:** *Forsythiae Fructus*, network pharmacology, molecular docking, hepatitis B virus, hepatocellular carcinoma, bicuculline

## Abstract

**Background:**

*Forsythiae Fructus* (FF), a widely used traditional Chinese medicine, possesses anti-inflammatory, antiviral, and anticancer properties. However, the precise anticancer mechanisms of FF against hepatitis B virus (HBV)-related hepatocellular carcinoma (HCC) remain poorly understood. This study therefore aims to investigate the therapeutic potential of FF in HBV-related HCC and elucidate its underlying mechanisms.

**Methods:**

The active components of FF and their putative target proteins were identified through network pharmacology, and their interactions were further validated via molecular docking and molecular dynamics (MD) simulations. *In vitro* assays were performed to evaluate the effects of FF extract on the viability, proliferation, and apoptosis of HBV-related HCC (HepG2.2.15) cells, along with the underlying molecular mechanisms. *In vivo* studies were performed to investigate the inhibitory effects of FF extract on subcutaneous xenograft tumors in nude mice, quantify serum cytokine levels, and evaluate the expression of key target proteins by immunohistochemistry.

**Results:**

A total of 23 active components of FF and their 201 associated targets were identified using the TCMSP database, whereas 1,296 differentially expressed genes related to HBV-related HCC were retrieved from the GEO database. We identified 42 overlapping target genes between FF and HBV-related HCC. KEGG pathway analysis revealed the IL-17 signaling pathway as a pivotal pathway, with three core genes (c-Jun, ESR1, and MMP9) demonstrating prognostic significance in survival outcomes. Ten compounds were classified as high-quality candidates. Molecular docking studies demonstrated that Bicuculline exhibited the strongest binding affinity toward the core target genes, while MD simulations confirmed the stability of Bicuculline-JUN/ESR1/MMP9 complexes. *In vitro* experiments demonstrated that FF extract significantly inhibited the viability and proliferation of HepG2.2.15 cells, induced apoptosis, and exerted its effects via modulation of the IL-17/MAPK signaling pathway. Notably, adenovirus-mediated overexpression experiments showed that ESR1 enhanced FF’s anti-HCC effects, whereas JUN and MMP9 partially counteracted them, confirming their roles as functional targets. *In vivo* studies further confirmed that FF suppressed tumor growth, reduced serum levels of ALT, AST, TNF-α, and IL-17B in mice, and modulated the expression of core target genes.

**Conclusions:**

The therapeutic potential of FF in HBV-related HCC was demonstrated, with its mechanism likely involving the regulation of multiple components, targets, and pathways. These findings establish a solid scientific foundation for exploring FF as a therapeutic option for HBV-related HCC.

## 1 Introduction

Hepatocellular carcinoma (HCC) accounts for approximately 80% of primary liver cancer cases and ranks as the third leading cause of cancer-related mortality worldwide (1). The principal risk factors associated with HCC include chronic hepatitis B virus(HBV) infection, alcoholic liver disease, and cirrhosis. HBV-related HCC specifically refers to HCC caused by HBV infection, with the Asia-Pacific region, particularly China, being recognized as a high-risk area (2, 3). It is noteworthy that over 80% of HCC cases are attributed to HBV infection (4), thereby making the pathogenesis and treatment of HBV-related HCC an active research focus (5). HBV exerts oncogenic effects through direct interactions and crosstalk with the host. Previous studies have demonstrated that integration of HBV gene fragments into human chromosomes can lead to abnormal expression and function of liver cell tumor-related genes (6). High-throughput sequencing data has revealed differential mutation rates in certain tumor-related genes between HBV-related HCC and other HCC cases (7). Due to the unique pathogenesis and complex biological processes involved in HBV-related HCC, treatment options for this type of cancer remain limited. Therefore, exploring new alternative therapeutic drugs and methods is still an urgent task.

The current first-line treatments for HBV-related HCC include surgical resection, liver transplantation, and systemic therapies such as tyrosine kinase inhibitors (e.g., sorafenib, lenvatinib) and immune checkpoint inhibitors (e.g., atezolizumab in combination with bevacizumab) (8, 9). Sorafenib, a multikinase inhibitor, targets VEGF receptors, PDGF receptors, and RAF kinases to suppress tumor angiogenesis and proliferation (10). Lenvatinib, another first-line option, inhibits VEGF receptors 1–3, FGFR 1–4, and other kinases, demonstrating non-inferiority to sorafenib in overall survival (11). Immune-based therapies like atezolizumab (anti-PD-L1) combined with bevacizumab (anti-VEGF) enhance antitumor immunity by blocking PD-1/PD-L1 interactions and normalizing tumor vasculature (12). Despite these advances, challenges such as drug resistance, adverse effects, and high recurrence rates persist, underscoring the need for alternative therapies (13).

Traditional Chinese medicine (TCM)-derived natural products and extracts have garnered attention as a potential reservoir of novel anti-cancer drugs due to their distinctive biological activities and minimal adverse effects (14). *Forsythiae Fructus* (FF), also known as Lianqiao in Chinese, refers to the desiccated fruit of *Forsythia suspensa* (Thunb.) Vahl, a member of the Oleaceae family (15, 16). The botanical nomenclature has been authenticated on World Flora Online (http://www.worldfloraonline.org/, accessed on November 2nd, 2024). FF has been extensively utilized in TCM for millennia and is regarded as one of the most fundamental botanicals, widely distributed across regions such as Shanxi, Henan, Shaanxi, and other regions of China (17). The FF was initially documented in the renowned ancient Chinese medical text “Shennong Bencao Jing” (Shen Nong’s Materia Medica) three centuries ago and has since gained widespread utilization as a traditional folk remedy for diverse ailments, encompassing heat clearance, detoxification, edema reduction, nodule dispersion, liver protection (18), and even anti-tumor effects. These therapeutic properties have been succinctly summarized in the 2020 edition of the Chinese Pharmacopoeia (19). In the Chinese Pharmacopoeia, there are 79 formulations that incorporate FF. It is frequently combined with Lonicerae Japonicae Flos in various formulations such as ‘Vitamin C and Forsythia Tablets’, ‘Double Yellow Lotus Granules’, and ‘Lianhua Qingwen Capsules’ (20, 21). Pharmacological investigations have revealed the presence of phenylethanol glycosides, forsythoside, pentacyclic triterpenes, and lignans in FF, which exhibit significant inhibitory effects against various types of cancer (22–24). However, due to the complex multi-target effect and multi-component nature of FF, its potential anti-tumor efficacy and underlying molecular mechanisms specifically targeting HBV-related HCC remain elusive.

Network pharmacology has emerged as a promising approach in recent years, integrating database mining, bioinformatics analysis, topological analysis, and molecular simulation to elucidate the intricate molecular mechanisms underlying TCM. Recent advancements in network pharmacology have significantly enhanced the understanding of TCM mechanisms, particularly in the context of complex multi-component systems like FF. For instance, studies have demonstrated that network pharmacology can effectively elucidate the synergistic effects of TCM compounds by integrating multi-target interactions and pathway analyses (25). This approach is especially valuable for identifying key bioactive components and their molecular targets in both single herbs and compound formulations (26). Furthermore, network pharmacology has been successfully applied to validate the therapeutic potential of TCM-derived monomers, bridging the gap between traditional use and modern drug discovery (27). This methodology is particularly advantageous for studying TCM due to its ability to map the ‘multi-component, multi-target, multi-pathway’ paradigm. This paradigm shift challenges the conventional mindset of ‘single disease-single target-single drug’ in drug development and is extensively employed for identifying potential therapeutic components from Chinese herbal medicines and predicting their plausible pharmacological mechanisms at the molecular level (28–30).

The ligand-receptor interaction theory-based molecular docking method is extensively employed in drug discovery to comprehend the binding and interaction of compounds with molecular targets, which holds immense significance for the development and application of active pharmaceutical ingredients (31, 32). Molecular dynamics (MD) simulations are a computational methodology that employs Newtonian mechanics to simulate and analyze the interactions between small molecules and proteins under diverse conditions (33). This approach assesses the stability and flexibility of binding interactions, thereby enabling the virtual screening of binding complexes and drug-target interactions with enhanced precision (34).

**Figure 1:**
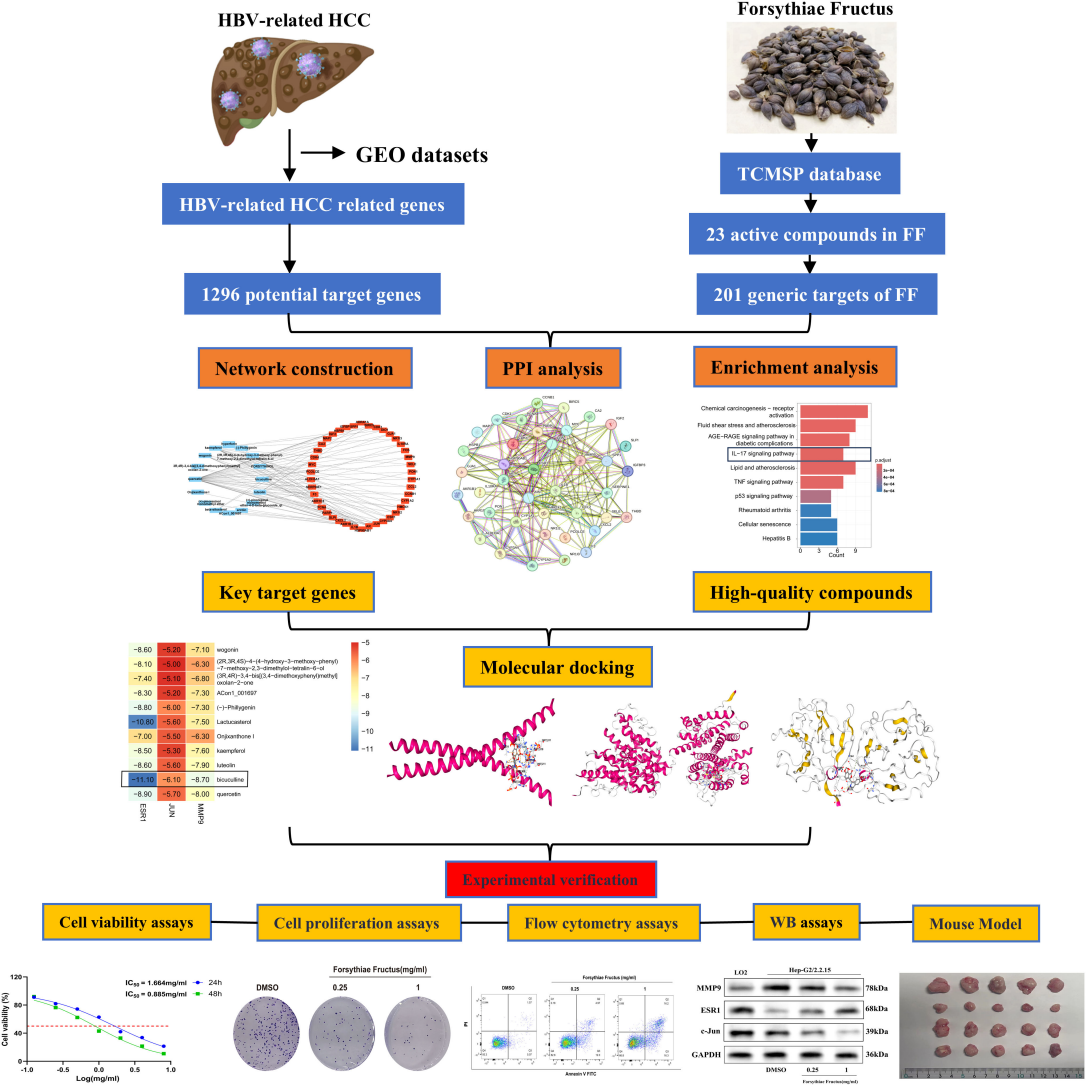
The workflow diagram of this study.

In this study, the active ingredients of FF were initially identified and screened via the Traditional Chinese Medicine Systems Pharmacology (TCMSP) database, followed by absorption, distribution, metabolism, excretion, and toxicity (ADMET) analysis to select drug-like compounds. Subsequently, using network pharmacology, the potential active ingredients, target genes, and signaling pathways of FF in treating HBV-related HCC were predicted. Lastly, molecular docking, MD simulations, and both *in vitro* and *in vivo* experiments were conducted to elucidate the key targets and molecular mechanisms underlying FF extract’s efficacy against HBV-related HCC. The overall workflow of this study is summarized in **Figure 1**. These findings may provide a theoretical foundation for FF clinical translation and suggest a potential therapeutic strategy for HBV-related HCC.

## 2 Materials and methods

### 2.1 Data collection and processing of HBV-related HCC

The study employed three GEO datasets (GSE47197, GSE55092, and GSE121248) obtained from the NCBI GEO database (https://www.ncbi.nlm.nih.gov/geo). Batch correction was performed using the ‘limma’ R package. A total of 180 HBV-related HCC samples and 191 non-tumor samples were included in the analysis. Data integration was conducted using the ‘sva’ R package. The identification of differentially expressed genes (DEGs) between HBV-related HCC and normal tissues was carried out using the ‘limma’ R package with |log FC| > 0.585 and adjusted P-value (adjust - P) < 0.05 as cutoff criteria for DEG identification.

### 2.2 Acquisition of active components and corresponding targets of FF

The FF component data is derived from the TCMSP database (https://old.tcmsp-e.com/tcmsp.php) (35). The selection of effective FF components primarily relies on their oral bioavailability (OB≥30%) and drug-likeness (DL≥0.18). Additionally, within the TCMSP database, effective component-target interactions are screened and target names are standardized to gene symbols using the UniProt database (https://www.uniprot.org/).

### 2.3 Construction of compound-target network and protein-protein interaction network

The ‘Venn’ R package was employed for the identification of common target genes in FF and HBV-related HCC. Subsequently, the Cytoscape_v3.9.1 software was utilized to construct an interaction network diagram encompassing the components and targets. To establish a protein-protein interaction (PPI) network among these overlapping targets, we referred to the STRING database (https://cn.string-db.org/) with a stringent criterion of an interaction score greater than 0.4.

### 2.4 Enrichment analysis of GO and KEGG pathways for common target genes

Gene Ontology (GO) and Kyoto Encyclopedia of Genes and Genomes (KEGG) enrichment analysis of the shared target genes was performed using the ‘Cluster Profiler’ and ‘Enrich plot’ packages in R software, with adjusted p-values < 0.05.

### 2.5 Identification and validation of key targets through comprehensive screening

The cytoHubba plugin in Cytoscape_v3.9.1 software was employed to identify core targets (36), followed by validation of their expression and prognostic relationship using the GEPIA (https://www.gepia.cancer-pku.cn) database (37).

### 2.6 Prediction of drug similarity using computer-based methods

Utilize the SwissADME web browser (http://www.swissdme.com) for conducting computer simulations to assess drug similarity among active compounds in FF (38). The evaluation of drug similarity is based on Lipinski’s five rules, wherein compounds failing to meet at least three criteria are deemed ineffective (39).

### 2.7 Molecular docking analysis

The SDF format files of high-quality compounds were obtained from the PubChem database (https://pubchem.ncbi.nlm.nih.gov/). The PDB format files of key target proteins (c-JUN, ESR1, and MMP9) were retrieved from the PDB database (https://www.rcsb.org/). Molecular docking simulations were conducted using the CB-Dock2 online tool (40).

### 2.8 Molecular dynamics simulation analysis

MD simulations were performed using Gromacs 2022 software. Force field parameters were generated using both the pdb2gmx tool (integrated in Gromacs) and the AutoFF web server. The CHARMM36 force field (41) was employed for receptor protein parameters, whereas ligand parameters were generated using the CGenFF force field. The system was solvated in a cubic TIP3P water box with a 1 nm buffer distance (42). The system was neutralized by adding appropriate counterions (Na+/Cl-) using the gmx genion tool with energy minimization. Long-range electrostatic interactions were calculated using the Particle Mesh Ewald method with a 10 Å cutoff distance. All bond lengths were constrained using the SHAKE algorithm, and integration steps of 1 fs were performed using the Verlet leapfrog algorithm. Prior to production runs, the system underwent energy minimization comprising 3000 steps of steepest descent optimization followed by 2000 steps of conjugate gradient minimization. This minimization protocol involved three sequential stages: (1) constrained solute with water minimization, (2) constrained counterions with minimization, and (3) full system minimization without constraints. Production simulations (100 ns) were performed in the isothermal-isobaric (NPT) ensemble at 310 K (37°C) using the Berendsen barostat. Throughout the simulations, standard Gromacs analysis tools were employed to calculate root-mean-square deviation (RMSD), root-mean-square fluctuation (RMSF), hydrogen bond formation, radius of gyration (Rg), and solvent-accessible surface area (SASA).

### 2.9 Preparation and extraction of FF

The FF was procured from Xiamen Hexiang Traditional Chinese Medicine Clinic affiliated with Beijing Tongrentang (Xiamen, China) and authenticated by Associate Professor Zhang Li from the Department of Traditional Chinese Medicine at Zhongshan Hospital affiliated to Xiamen University. The botanical classification and species identification of the tested plant have been ascertained, and the samples have been archived in the Specimen Museum of Fujian Key Laboratory for Chronic Liver Disease and Hepatocellular Carcinoma at Zhongshan Hospital affiliated to Xiamen University with the assigned specimen number 19. 100g of FF was weighed and soaked overnight in ultra-pure water at a ratio of ten parts water to one part herb. Subsequently, the soaked FF underwent two rounds of boiling at 100°C for 60 minutes each time, followed by cooling and filtration. The resulting filtrate was combined and concentrated to an appropriate volume before being stored overnight at -20°C. On the following day, freeze-drying of the FF powder was conducted using a freeze dryer (Telstar, Japan) set at -20°C. The resultant powder was then preserved for future use. For cell experiments, 100 mg of FF powder was dissolved in 1 mL of ultra-pure water and thoroughly vortexed. After centrifugation at 10,000 rpm for 10 minutes and sterilization through a 0.22 μm filter, it was stored accordingly. The original solution with a concentration of 100 mg/mL could be diluted as required in buffer or culture medium based on experimental needs.

### 2.10 Cell lines and reagents

The HepG2.2.15 cell line was selected for this study due to its unique relevance to HBV-related HCC. Derived from HepG2 cells stably transfected with a recombinant plasmid containing the full-length HBV genome, HepG2.2.15 cells consistently produce HBV virions and express HBV antigens, making them a clinically relevant model for investigating HBV-related HCC (43). This cell line recapitulates key features of HBV infection, including viral replication and integration, which are critical for studying the molecular mechanisms of HBV-related HCC (44). Additionally, HepG2.2.15 cells retain the tumorigenic properties of parental HepG2 cells, such as proliferation and invasion capabilities, thereby providing a robust platform to evaluate the anti-tumor effects of FF extract. The HepG2.2.15 cells were obtained from the Shanghai Cell Bank of the Chinese Academy of Sciences and cultured in MEM medium supplemented with 10% fetal bovine serum and 0.38ug/mL G418 at a temperature of 37°C with a CO2 concentration of 5%. Upon reaching 80% cell growth density, passaging was conducted, and logarithmic phase cells were utilized for experimental investigations. For Western blot analysis, the following primary antibodies were employed: Anti-GAPDH antibody (ab8245, Abcam), Anti-c-Jun antibody (ab40766, Abcam), Anti-Estrogen Receptor alpha antibody (21244-1-AP, Proteintech), Anti-MMP9 antibody (ab283575, Abcam), Anti-IL-17RB antibody (ab86488, Abcam), phosphorylated p38 antibody(catalog#4511, Thr180/Tyr182, CST); p38 MAPK antibody(catalog#9212, CST); phosphorylated ERK1/2 antibody(catalog#4370, Thr202/Tyr204, CST); ERK1/2 antibody(catalog#4695, CST).

### 2.11 Cell transfection

The recombinant adenoviruses ESR1-OE and ESR1-NC, JUN-OE and JUN-NC, MMP9-OE and MMP9-NC were provided by Genechem Company (Shanghai, China). Specifically, the ESR1-OE, JUN-OE, and MMP9-OE adenoviruses were designed to achieve the overexpression of ESR1, JUN, and MMP9, respectively, while ESR1-NC, JUN-NC, and MMP9-NC served as their corresponding negative controls. These adenoviruses were transfected into HepG2.2.15 cells for 8 hours, followed by selection of successfully transfected cells using puromycin (3 μg/mL). Finally, the transfection efficiency was confirmed by Western blot analysis.

### 2.12 50% inhibiting concentration and cell proliferation assay

The HepG2.2.15 cells were seeded at a density of 5 × 10^3^ cells per well in a 96-well microplate, followed by exposure to varying concentrations of FF for either 24 or 48 hours. Subsequently, the culture medium was replaced and the results were assessed using Cell Counting Kit-8 (CCK-8) (#PF00004, Proteintech). The OD values were determined using a microplate reader (VT, USA) at a wavelength of 450 nm. IC_50_ values were determined through nonlinear regression analysis.

Dilute the pre-treated cells (either drug intervention or transfection) with DMEM medium to a final concentration of 5 × 10^4 cells/mL. Add 100 μL of the cell suspension to each well of a 96-well plate and incubate the cells at 37°C with 5% CO2 for varying durations (0, 1, 2, 3, 4, and 5 days). At the end of each designated incubation period, add the CCK-8 reagent and incubate for an additional hour. Finally, measure the OD values at 450 nm to assess the relative changes in cell proliferation across the specified time intervals.

### 2.13 Colony formation assay

The HepG2.2.15 cells were seeded overnight in a 12-well plate at a density of 500 cells per well and subsequently exposed to varying concentrations of FF for treatment purposes. Post-treatment, the cells underwent incubation within a CO2 incubator set at 37°C for a duration ranging from 7 to 14 days while ensuring that complete medium was replenished every 3 days. Fixation of cell colonies occurred on either day fourteen or upon reaching ≥50 colonies by employing a fixative solution consisting of 4% paraformaldehyde (PFA), followed by staining using an aqueous solution comprising of crystal violet (0.1%, #C0121-100mL, Beyotime).

### 2.14 Detection of cell apoptosis

The HepG2.2.15 cells were treated with various concentrations of FF for 48 hours. Following the guidelines provided by the manufacturer (YEASEN, #40305ES20, China), adherent and free-floating cells were collected and subjected to staining using fluorescent dyes FITC-Annexin V and PI. Subsequently, flow cytometry analysis was performed using a BD LSRFortessa™X-20 instrument (USA).

### 2.15 Western blot analysis

HepG2.2.15 cells were subjected to treatment with varying concentrations of drugs for a duration of 48 hours, followed by lysis in RIPA lysis buffer (#PR20035, Proteintech) to facilitate the extraction of total proteins. Equal amounts of protein were separated by SDS-PAGE (10%) and transferred onto a PVDF membrane (Millipore, Billerica, MA, USA). The PVDF membrane was blocked with 5% skim milk in TBST at room temperature for 1 hour. Subsequently, the membrane was incubated overnight at 4°C with the specific primary antibody. After three washes of 10 minutes each with TBST, the membrane was incubated for 1 hour with the secondary antibody solution. Finally, enhanced chemiluminescence reagent (ECL) (#34580, Thermo Scientific) was used for incubation and visualization of protein bands using a chemiluminescence imaging system (Tanon 5200, Shanghai, China).

### 2.16 Animals and experimental design

Male BALB/c nude mice (n = 20) were obtained from the Animal Experiment Center of Xiamen University. The mice were housed in an SPF-level animal facility and acclimatized to the environment for one week. Each mouse received a subcutaneous injection of 5 × 10^6 HepG2.2.15 cells (100 μL in PBS) near the axilla on the back. One week post-injection, the mice were randomly assigned into four groups (n = 5 per group). Group assignments were as follows: Model group: daily intragastric administration of normal saline; Positive control group: daily intragastric administration of sorafenib at 60 mg/kg; FF group: intragastric administration of FF extract at 5 g/kg or 10 g/kg every other day. The intragastric route was chosen because: (1) it replicates the clinical oral administration of FF in traditional medicine practice (16); (2) previous pharmacokinetic studies confirmed gastrointestinal absorption of FF’s key components like forsythoside A and phillyrin (17); and (3) it minimizes stress in immunocompromised nude mice compared to invasive routes (22). Treatment duration was two weeks. Tumor dimensions were measured every other day using a vernier caliper, and tumor volume was calculated using the formula: Volume = 1/2 × (length × width^2). On day 15, blood samples (0.5 mL per mouse) were collected via orbital puncture for further analysis. Subsequently, all mice were euthanized, and tumor tissues were excised and weighed for hematoxylin and eosin (H&E) staining and immunohistochemistry (IHC) staining. All animal care and use procedures were approved by the Ethics Committee of Zhongshan Hospital Affiliated to Xiamen University (Approval Number: xmzsyyky-2024-621).

### 2.17 H&E staining

Mouse tumor tissues were fixed, embedded, and sectioned. Following dewaxing and a 2-minute water wash, the sections were stained with hematoxylin for 5 to 10 minutes, rinsed with running water, and subsequently immersed in eosin solution for 1 to 3 minutes, followed by another rinse with running water. The sections then underwent conventional dehydration, clearing, mounting, and cover slipping. Microscopic examination was performed, and images were captured.

### 2.18 Immunohistochemistry assay

IHC assay was conducted to evaluate the expression of core targets in tumor tissues. Paraffin-embedded mouse tumor samples were subjected to IHC staining. Specifically, 4 μm thick sections were prepared and dewaxed, followed by quenching of endogenous peroxidase activity. Sections were then blocked with normal goat serum and incubated overnight at 4°C with primary antibodies against c-Jun, MMP9, and ESR1. After three washes with PBS, the sections were incubated with HRP-conjugated secondary antibodies at room temperature. The sections were counterstained with hematoxylin, dehydrated using ethanol and xylene, and mounted. Images were captured using a Hamamatsu optical microscope at a magnification of ×200. Five non-overlapping fields of view were randomly selected for each slice at 200x magnification, and the average optical density(AOD) value was calculated by image J software.

### 2.19 Determination of serum biochemical indicators

The levels of ALT, AST, TNF-α, and IL-17B in mouse serum were quantitatively measured using biochemical assay kits. Samples were processed according to the manufacturer’s instructions and analyzed via enzyme-linked immunosorbent assay (ELISA).

### 2.20 Statistical analysis

Statistical analysis was performed using GraphPad Prism 10 software (CA, USA) and R software version 4.4.2. The results were reported as mean ± standard deviation. Group differences were assessed using Student’s t-tests and one-way analysis of variance(ANOVA). Kaplan-Meier method was used for OS analysis. A p-value < 0.05 indicated statistical significance.

## 3 Results

### 3.1 Network pharmacology analysis reveals multi-target mechanisms of FF in HBV-related HCC

**Figure 2:**
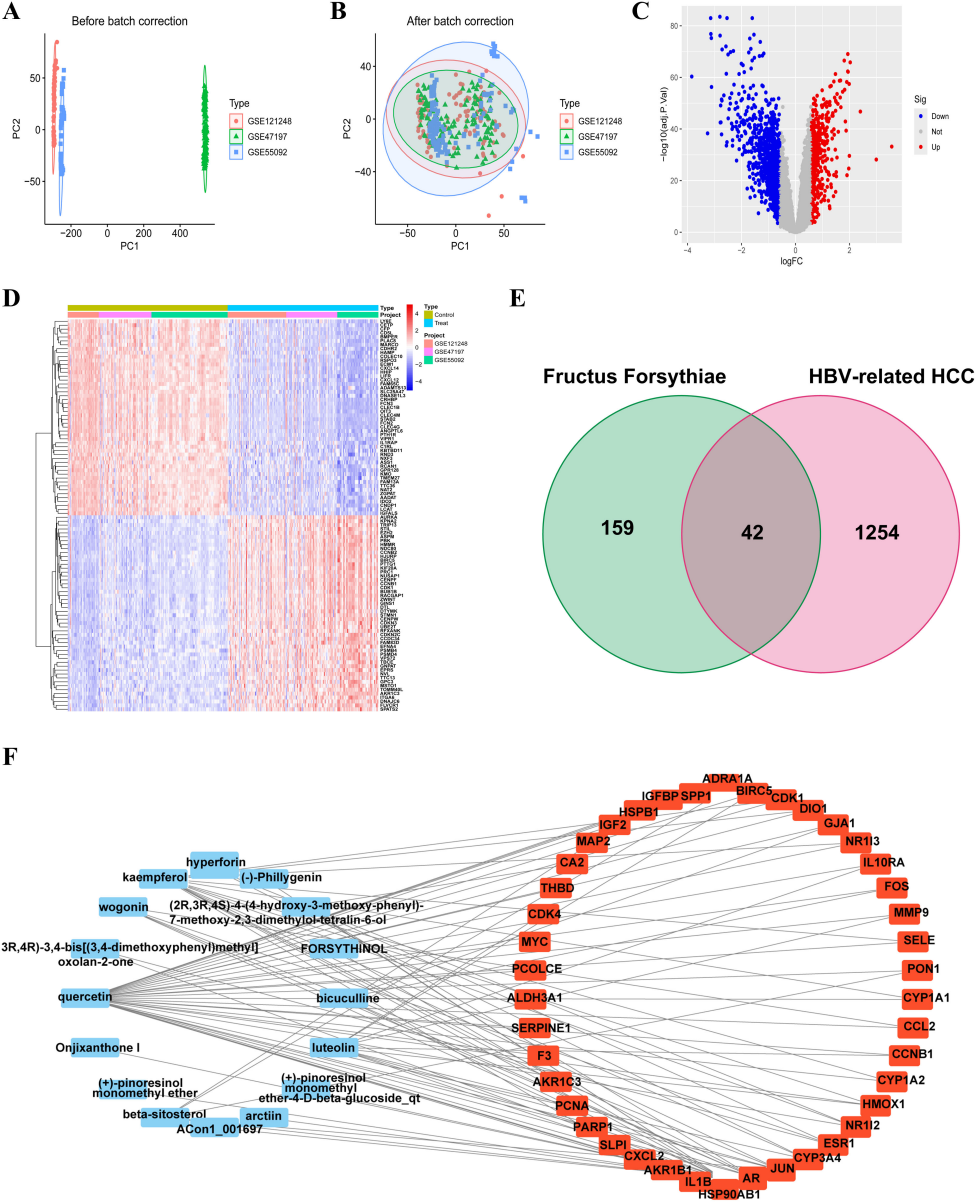
Identification and construction of the interaction network between FF and HBV-related HCC. Merge and rectify three datasets related to HBV-associated HCC **(A)** prior to correction. **(B)** post-correction. **(C)** The volcano plot of differentially expressed genes in HBV-related HCC is presented, with red dots indicating significantly upregulated genes and blue dots representing significantly downregulated genes. **(D)** The expression of differentially expressed genes in the normal group and the tumor group was characterized by high expression in red and low expression in blue. **(E)** The Venn diagram illustrates the shared target genes between FF and HBV-related HCC. **(F)** Relationship between the active ingredients of FF and the common targets of FF and HBV-related HCC. FF, *Forsythiae Fructus*; HBV-related HCC, hepatitis B virus-related hepatocellular carcinoma.

Principal component analysis (PCA) of the integrated GEO datasets (GSE47197, GSE55092, and GSE121248) confirmed successful batch effect correction (**Figures 2A, B**). Subsequently, differential expression analysis identified 1,296 significantly dysregulated genes (857 downregulated and 439 upregulated) in HBV-related HCC compared to non-tumor tissues (**Figures 2C, D**). From the TCMSP database, 23 bioactive compounds in FF met screening criteria (OB ≥30%, DL ≥0.18), spanning several important phytochemical classes such as terpenoids exemplified by β-amyrin acetate, lignans including phillyrin, flavonoids represented by quercetin and kaempferol, and alkaloids notably bicuculline (**Table 1**). A total of 201 target genes associated with HBV-related HCC were retrieved from the TCMSP database. Using the Venn diagram tool, we identified 42 overlapping target genes between FF and HBV-related HCC (**Figure 2E**). Subsequently, a component-disease target interaction analysis was performed (**Figure 2F**), revealing that 16 FF-derived compounds may specifically target these 42 shared genes. These findings highlight the diverse bioactive compounds in FF that may have therapeutic potential against HBV-related HCC.

**Table 1:** Information on active compounds of *Forsythiae Fructus* screened by TCMSP.

Herb	MOL ID	Compound name	OB (%)	DL
Lianqiao	MOL000173	wogonin	30.68	0.23
Lianqiao	MOL003281	20(S)-dammar-24-ene-3β,20-diol-3-acetate	40.23	0.82
Lianqiao	MOL003283	(2R,3R,4S)-4-(4-hydroxy-3-methoxy-phenyl)-7-methoxy-2,3-dimethylol-tetralin-6-ol	66.51	0.39
Lianqiao	MOL003290	(3R,4R)-3,4-bis[(3,4-dimethoxyphenyl)methyl]oxolan-2-one	52.3	0.48
Lianqiao	MOL003295	(+)-pinoresinol monomethyl ether	53.08	0.57
Lianqiao	MOL003305	PHILLYRIN	36.4	0.86
Lianqiao	MOL003306	ACon1_001697	85.12	0.57
Lianqiao	MOL003308	(+)-pinoresinol monomethyl ether-4-D-beta-glucoside_qt	61.2	0.57
Lianqiao	MOL003315	3beta-Acetyl-20,25-epoxydammarane-24alpha-ol	33.07	0.79
Lianqiao	MOL000211	Mairin	55.38	0.78
Lianqiao	MOL003322	FORSYTHINOL	81.25	0.57
Lianqiao	MOL003330	(-)-Phillygenin	95.04	0.57
Lianqiao	MOL003344	β-amyrin acetate	42.06	0.74
Lianqiao	MOL003347	hyperforin	44.03	0.6
Lianqiao	MOL003348	adhyperforin	44.03	0.61
Lianqiao	MOL003365	Lactucasterol	40.99	0.85
Lianqiao	MOL003370	Onjixanthone I	79.16	0.3
Lianqiao	MOL000358	beta-sitosterol	36.91	0.75
Lianqiao	MOL000422	kaempferol	41.88	0.24
Lianqiao	MOL000522	arctiin	34.45	0.84
Lianqiao	MOL000006	luteolin	36.16	0.25
Lianqiao	MOL000791	bicuculline	69.67	0.88
Lianqiao	MOL000098	quercetin	46.43	0.28

### 3.2 Integrated network pharmacology analysis reveals key pathways and functional annotations in HBV-related HCC treatment

**Figure 3:**
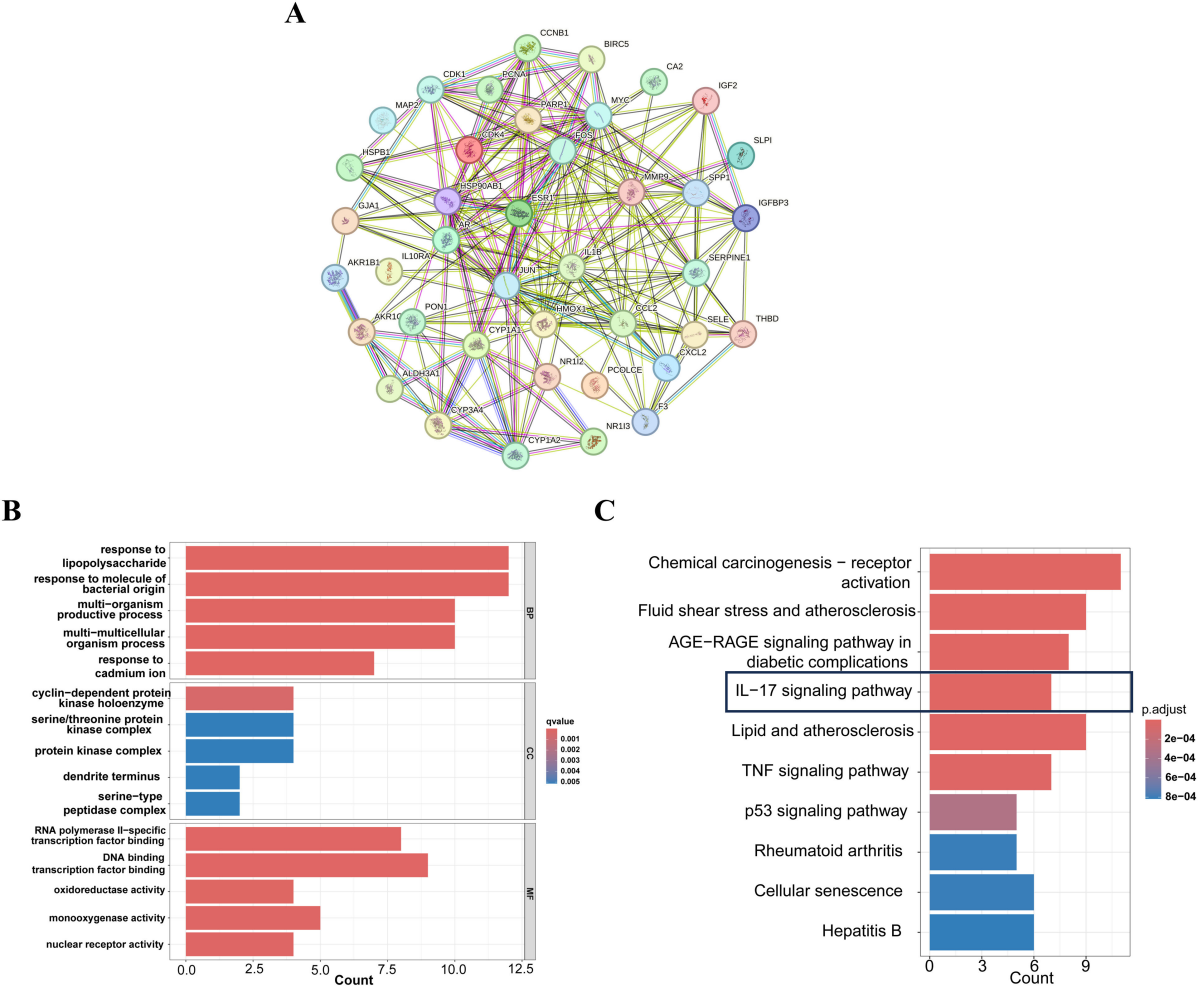
PPI,GO and KEGG enrichment analysis of FF-HBV-related HCC targets. **(A)** Protein-protein interaction (PPI) network of shared target genes between FF and HBV-related HCC. **(B)** GO (Biological process, molecular function, and cellular component) analysis (top 5). The node length represents the number of target genes enriched, and the node color from blue to red represents the q-value from large to small. **(C)** KEGG pathway enrichment analysis (top 10). The node size represents the number of target genes enriched, and the node color from blue to red represents the q-value from large to small. FF: *Forsythiae Fructus*; HBV-related HCC: hepatitis B virus-related hepatocellular carcinoma.

A comprehensive analysis of the interactions among the 42 common target genes was performed through PPI network analysis (**Figure 3A**). After removing non-interacting targets, the PPI network consisted of 40 nodes and 228 edges. GO analysis demonstrated that the shared target genes were most significantly associated with responses to lipopolysaccharide, bacterial molecules, and multi-organism reproductive processes. The predominant cellular components included cyclin-dependent protein kinase holoenzyme complex, serine/threonine protein kinase complex, and protein kinase complex. The key molecular functions were RNA polymerase II-specific transcription factor binding, DNA-binding transcription factor binding, and oxidoreductase activity (**Figure 3B**). KEGG pathway enrichment analysis identified the top 10 significantly enriched pathways in FF-treated HBV-related HCC, including Chemical carcinogenesis-receptor activation, Fluid shear stress and atherosclerosis, AGE-RAGE signaling pathway in diabetic complications, IL-17 signaling pathway, and Lipid and atherosclerosis (**Figure 3C**). These results demonstrate complex interactions among the shared target genes associated with FF treatment of HBV-related HCC, involving diverse biological processes and signaling pathways.

### 3.3 Comprehensive screening identifies key therapeutic targets and bioactive compounds

**Figure 4:**
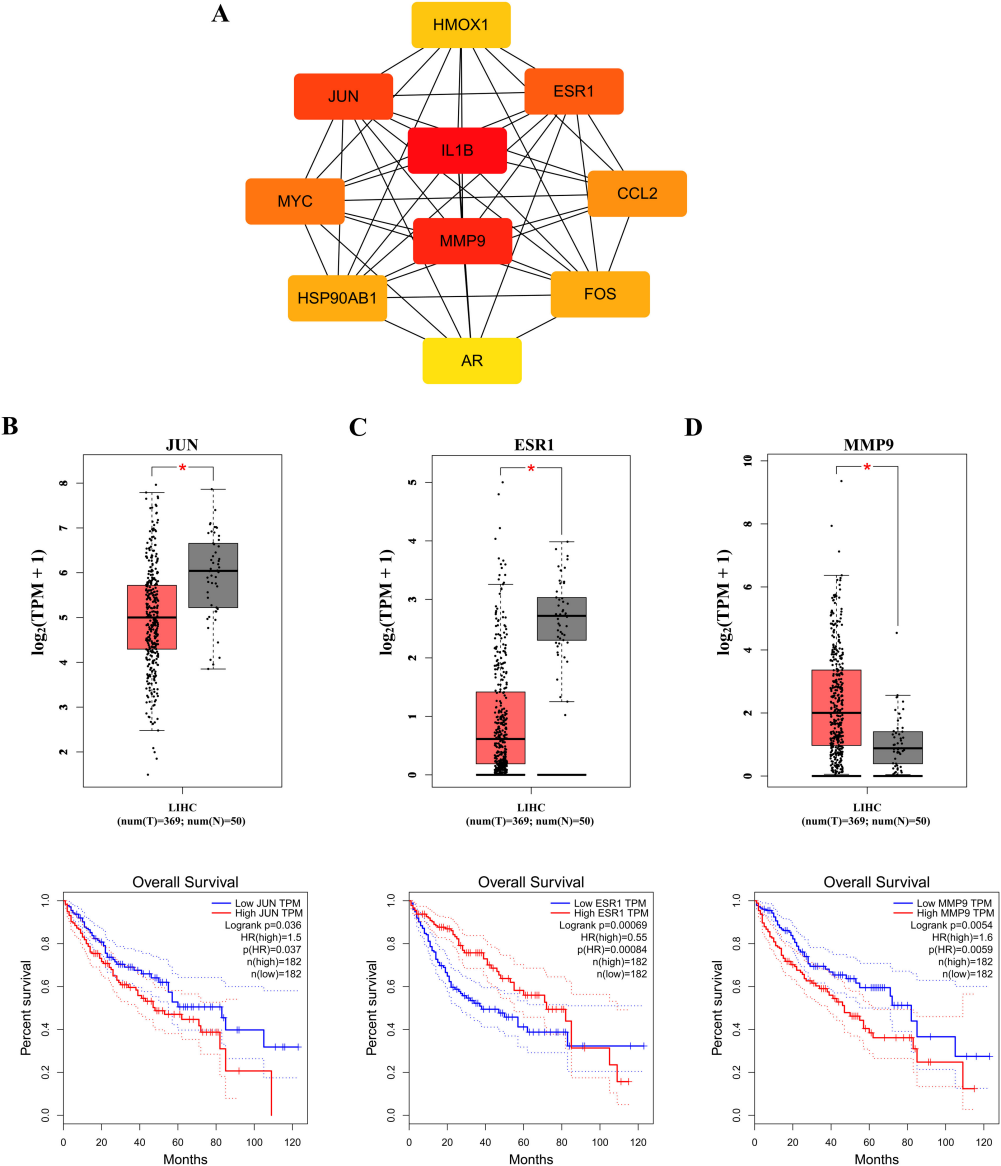
Identification of the core targets. **(A)** The Top 10 hub genes. Expression and prognostic analysis of three key genes in relation to survival outcomes in the GEPIA TCGA-LIHC datasets: **(B)** JUN gene; **(C)** ESR1 gene and **(D)** MMP9 gene. HR, hazard risk. *p < 0.05.

To identify core therapeutic targets for FF in HBV-related HCC treatment, we performed network centrality analysis using the CytoHubba plugin in Cytoscape on the 42 shared target genes, identifying the top 10 hub targets (**Figure 4A**). The topological parameters (degree centrality and betweenness centrality) for these hub genes are summarized in **Table 2**. These 10 hub targets underwent additional validation through differential expression and survival analysis. Analysis of the TCGA-LIHC dataset via GEPIA revealed that only JUN, ESR1, and MMP9 showed both significant differential expression and prognostic relevance (**Figures 4B-D**). These findings suggest that JUN, ESR1, and MMP9 play crucial roles in HCC pathogenesis and progression, warranting further investigation.

**Table 2:** Top ten targets information of PPI network.

Gene	Degree	Betweenness Centrality
IL1B	28	273.85685
MMP9	25	120.33292
JUN	24	92.27915
ESR1	23	100.29104
MYC	21	69.59881
CCL2	20	142.47596
FOS	19	46.92628
HSP90AB1	19	89.98149
HMOX1	17	39.2768
AR	16	37.74052

We next evaluated the drug-likeness of FF’s active compounds using Lipinski’s rule and ADMET predictions. **Table 3** presents 10 compounds with favorable drug-like properties, after excluding 3 compounds lacking PubChem entries and 3 failing ADMET criteria, confirming their pharmaceutical potential.

**Table 3:** Drug-likeness prediction of *Forsythiae Fructus* ingredients by ADMET evaluation using SwissADME software.

Compound Name	MW(g/mol)	HBA	HBD	RB	TPSA (Å2)	Lipinski’s Rule	GI Absorption	BBB	Solubility	ADMET Screening
wogonin	284.26	5	2	2	79.9	Yes	High	No	Moderately soluble	Yes
(2R,3R,4S)-4-(4-hydroxy-3-methoxy-phenyl)-7-methoxy-2,3-dimethylol-tetralin-6-ol	360.4	6	4	5	99.38	Yes	High	No	soluble	Yes
(3R,4R)-3,4-bis[(3,4-dimethoxyphenyl)methyl] oxolan-2-one	386.44	6	0	8	63.22	Yes	High	Yes	Moderately soluble	Yes
ACon1_001697	372.41	6	1	5	66.38	Yes	High	Yes	soluble	Yes
(-)-Phillygenin	372.41	6	1	5	66.38	Yes	High	Yes	soluble	Yes
hyperforin	536.78	4	1	11	71.44	No	Low	No	Poorly soluble	No
Onjixanthone I	302.28	6	1	3	78.13	Yes	High	Yes	soluble	Yes
beta-sitosterol	414.71	1	1	6	20.23	Yes	Low	No	Poorly soluble	No
kaempferol	286.24	6	4	1	111.13	Yes	High	No	soluble	Yes
arctiin	534.55	11	4	10	153.37	No	Low	No	soluble	No
luteolin	286.24	6	4	1	111.13	Yes	High	No	soluble	Yes
bicuculline	367.35	7	0	1	66.46	Yes	High	Yes	soluble	Yes
quercetin	302.24	7	5	1	131.36	Yes	High	No	soluble	Yes

MW, molecular weight; HBA, hydrogen bond acceptors; HBD, hydrogen bond donors; RB, rotatory bonds; GI, gastrointestinal absorption; BBB, blood brain barrier.

### 3.4 Investigation of molecular docking

**Figure 5:**
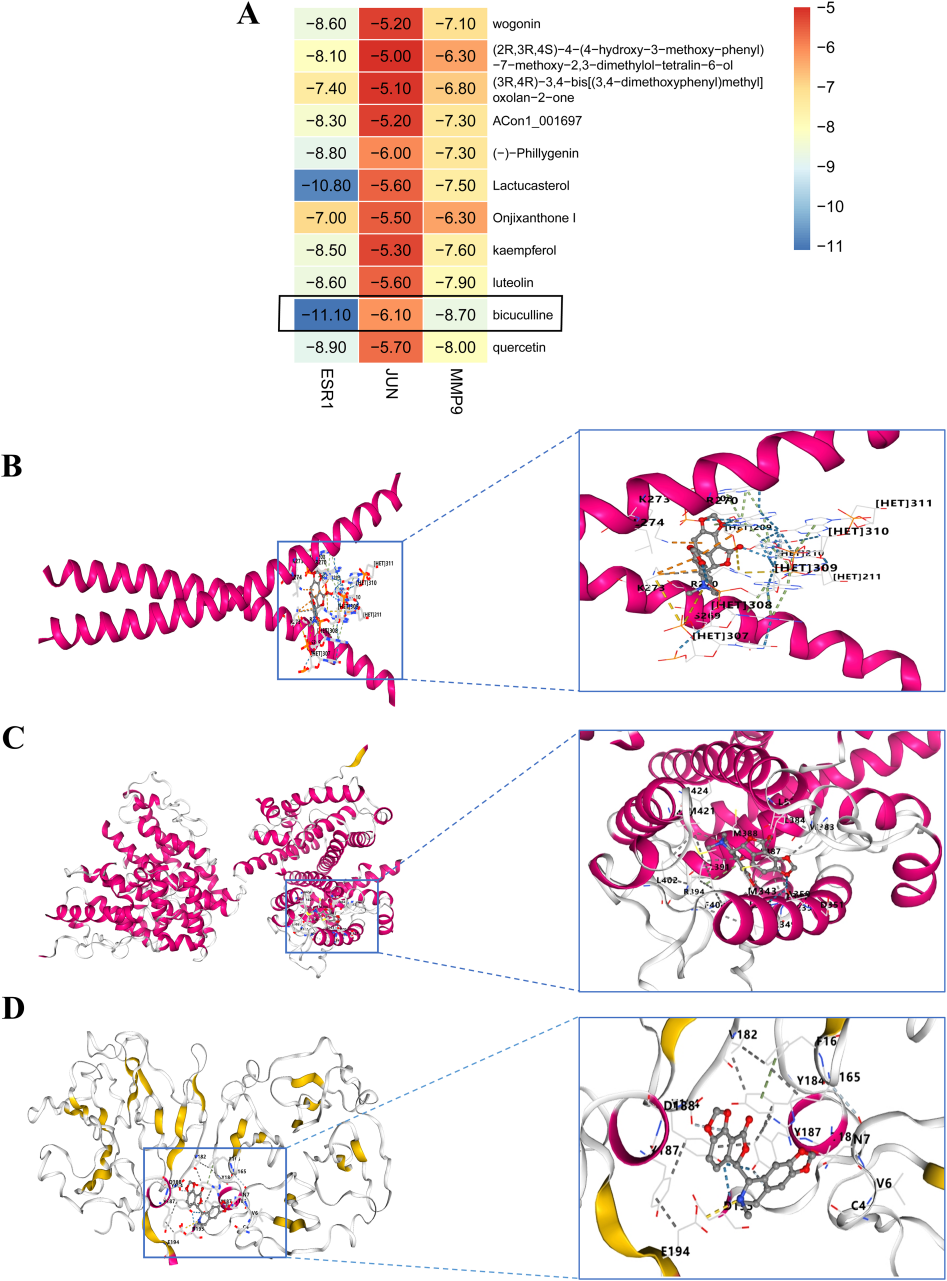
Molecular docking outcomes of high-quality compounds in FF were analyzed. **(A)** The heat map illustrates the results of molecular docking for 10 high-quality compounds targeting three core genes. **(B-D)** The results of 3D docking were obtained by combining with the three most active docking pairs, namely Bicuculline and JUN, Bicuculline and ESR1, as well as Bicuculline and MMP9. FF, *Forsythiae Fructus*.

We performed molecular docking analysis of the 10 bioactive compounds against the three core targets (JUN, ESR1, and MMP9), with binding affinities visualized in **Figure 5A**. All compounds demonstrated strong binding affinities (<-5 kcal/mol) with the target proteins, suggesting stable ligand-receptor interactions (45). Notably, bicuculline showed the strongest binding affinity for JUN, ESR1, and MMP9 (**Figures 5B-D**), highlighting its potential as a lead compound in FF’s anti-HBV-related-HCC activity.

### 3.5 Molecular dynamics simulations between the core targets and bicuculline

The RMSD serves as a reliable metric for evaluating the conformational stability of protein-ligand complexes and quantifying the positional deviation of atoms from their initial coordinates. Lower RMSD values indicate greater conformational stability. RMSD analysis revealed that the JUN-Bicuculline complex reached equilibrium after 80 ns, exhibiting stable fluctuations around 2.82 Å. Similarly, the ESR1-Bicuculline system reached equilibrium at 30 ns with final oscillations at 3.4 Å, while the MMP9-Bicuculline complex stabilized after 85 ns, fluctuating around 4.2 Å (**Supplementary Figure S1A**). These findings suggest strong binding stability between bicuculline and all three target proteins. Rg analysis reflected the structural compactness of the complexes, with larger Rg variations suggesting potential system expansion. Notably, all three complexes showed minimal Rg fluctuations during the simulation (**Supplementary Figure S1B**), indicating negligible structural expansion or contraction. SASA analysis showed moderate fluctuations prior to stabilization in all complexes (**Supplementary Figure S1C**), suggesting bicuculline-induced microenvironmental changes at the binding interfaces.

Hydrogen bond (HBond) analysis indicated dynamic interactions: the JUN-Bicuculline complex maintained an average of one HBond (range: 0–3), whereas ESR1-Bicuculline and MMP9-Bicuculline complexes each sustained approximately two HBonds (ranges: 0–5 and 0–6, respectively; **Supplementary Figure S1D**). These results confirm stable hydrogen bonding networks between the ligand and target proteins. Residue flexibility analysis through RMSF showed low fluctuation values (mostly 0.7–2.1 Å) for all complexes (**Supplementary Figures S1E-G**), indicating restricted backbone mobility and high structural stability.

In conclusion, the JUN-Bicuculline, ESR1-Bicuculline, and MMP9-Bicuculline complexes exhibit stable binding, supported by consistent hydrogen bonding interactions. Thus, bicuculline demonstrates favorable binding affinity for the target proteins JUN, ESR1, and MMP9.

### 3.6 The effects of FF extract on the viability, proliferation, and apoptosis of HepG2.2.15 cells and its potential molecular mechanisms

**Figure 6:**
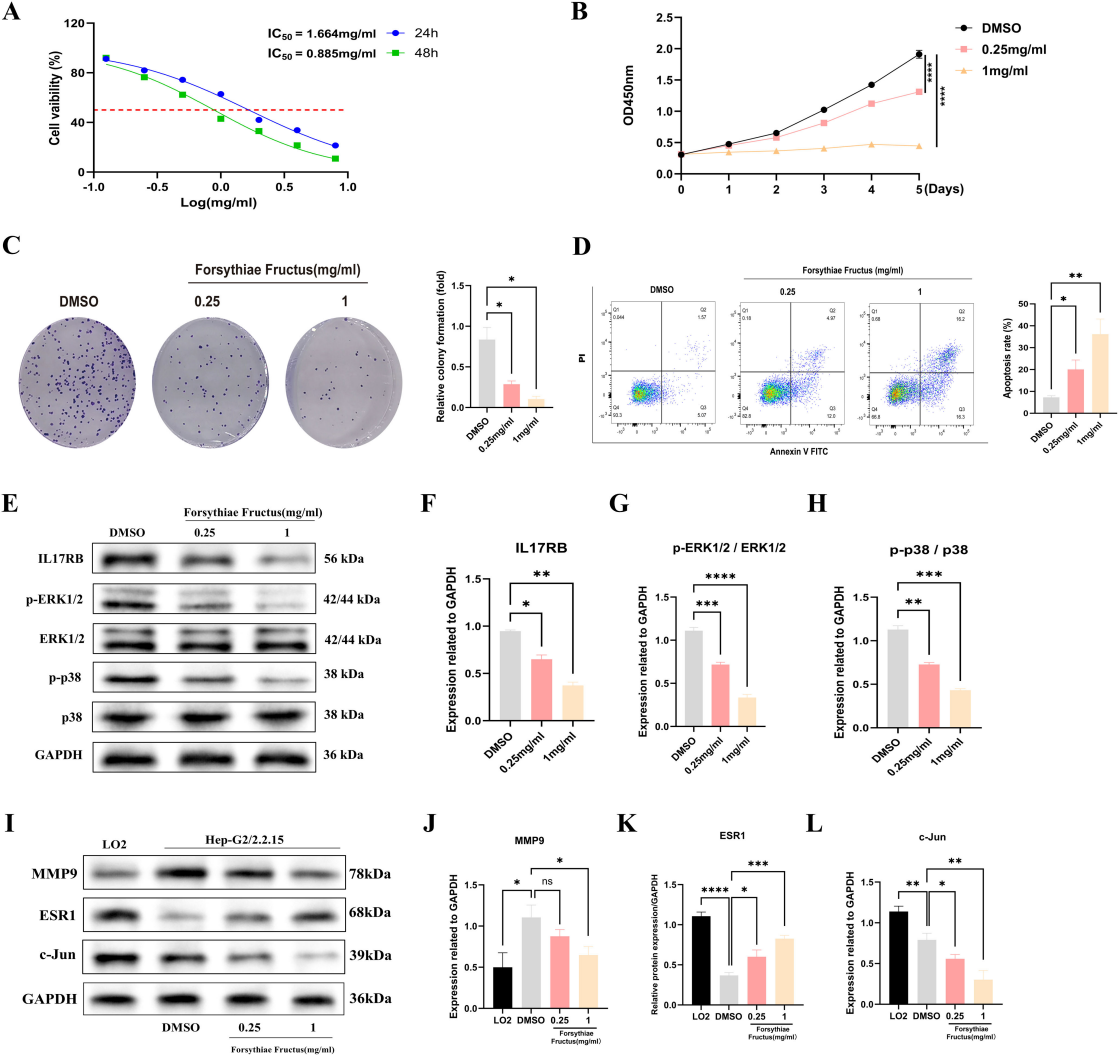
FF significantly affected the viability, proliferation and apoptosis of HBV-related HCC cells and its molecular mechanism. **(A)** The viability of HepG2.2.15 cells was measured by CCK8 assays following treatment with FF extract for 24 and 48 h. **(B)** Following treatment of HepG2.2.15 cells with FF extract at various concentrations, cell proliferation was assessed from day 1 to day 5 using the CCK-8 assay. **(C)** Effect of FF extract on HepG2.2.15 cells clonogenic ability. **(D)** Flow cytometry was used to evaluate the induction of apoptosis in HepG2.2.15 cells after treatment with FF extract at different concentrations for 48 hours. **(E)** After treating HepG2.2.15 cells with FF extracts of different concentrations for 48 hours, the expression of relevant proteins was detected by Western blotting. **(F-H)** Quantitative analysis was conducted to assess the expression of IL-17 protein, as well as the levels of ERK1/2 and p38 protein phosphorylation, following treatment with various concentrations of FF extract. **(I)** The expression of MMP9, ESR1, and c-Jun proteins in normal liver cells (LO2) and HBV-related HCC cells (HepG2.2.15), as well as their expression after treatment with different concentrations of FF for 48 hours, was examined using Western blot analysis. **(J-L)** Quantitative analysis of MMP9, ESR1, and c-Jun protein expression in normal liver cells (LO2) and HBV-related HCC cells (HepG2.2.15), as well as the subsequent changes in expression following treatment with varying concentrations of FF in HepG2.2.15 cells. FF: *Forsythiae Fructus*. All data are presented as the means ± S.D., *p < 0.05, **p < 0.01, ***p < 0.001, ****p < 0.0001, ns: no significant difference.

To investigate the impact of FF extract on HBV-related HCC cells, we conducted subsequent experimental studies using HepG2.2.15 cells, a well-established *in vitro* model for HBV-related HCC research due to their derivation from HepG2 cells transfected with a recombinant plasmid containing the full-length HBV-DNA gene (43). The CCK-8 assay revealed a concentration- and time-dependent decrease in the viability of HepG2.2.15 cells following FF extract treatment (**Figure 6A**). The IC50 values were calculated as 1.664 mg/mL at 24 h and 0.885 mg/mL at 48 h. Furthermore, CCK-8 analysis confirmed the dose-dependent inhibitory effect of FF extract on HepG2.2.15 cell proliferation (**Figure 6B**). Consistently, the colony formation assay demonstrated a significant reduction in colony-forming ability after FF extract treatment (**Figure 6C**), reinforcing its anti-proliferative potential. Finally, flow cytometry was used to measure the total apoptotic population. Although late apoptosis alone was not prominently observed, the overall apoptotic population significantly increased in a FF dose-dependent manner (**Figure 6D**), which is consistent with the anti-proliferative effects detected in viability and colony formation assays. These findings suggest that FF exhibits promising potential for the treatment of HBV-related HCC.

The KEGG analysis revealed that the significantly enriched common target genes were primarily associated with the IL-17 signaling pathway, suggesting its potential molecular mechanism in FF-mediated treatment of HBV-related HCC. Previous studies have demonstrated that IL-17RB, a key receptor in the IL-17 signaling pathway, is closely associated with tumor initiation and progression (46–48). IL-17RB activates downstream signaling pathways, promoting tumor cell proliferation, invasion, and metastasis. Western blot results demonstrated that FF extract significantly suppressed IL-17RB expression and the phosphorylation of downstream signaling molecules, including ERK1/2 and P38 MAPK (**Figures 6E-H**). These results indicate that FF inhibits HBV-related HCC by modulating the IL-17RB/MAPK signaling pathway. We further validated FF’s targeting effect on the three core genes via Western blot. Compared to normal LO2 hepatocytes, HepG2.2.15 cells exhibited significantly lower c-Jun and ESR1 expression but markedly higher MMP9 levels. Furthermore, increasing FF concentrations dose-dependently upregulated ESR1 expression but downregulated c-Jun and MMP9 levels in HepG2.2.15 cells (**Figures 6I-L**). These findings suggest that FF modulates the expression of these core target genes.

To further elucidate the functional relationship between FF and core genes, we utilized recombinant adenoviruses to overexpress three candidate genes individually. Successful transfection was verified by assessing the protein expression levels of ESR1, JUN, and MMP9 via Western blot analysis (**Supplementary Figures S2A-C**). Next, we employed the CCK-8 assay to assess the impact of FF extract at its half maximal inhibitory concentration (0.885 mg/mL) on cell proliferation capacity under conditions where core genes were overexpressed. As shown in **Supplementary Figure S2D**, ESR1 overexpression significantly inhibited HepG2.2.15 cell viability and enhanced their sensitivity to FF extract. In contrast, JUN overexpression markedly increased HepG2.2.15 cell viability and partially counteracted the FF extract-induced suppression of proliferation (**Supplementary Figure S2E**). Although MMP9 overexpression did not significantly increase HepG2.2.15 cell viability, it slightly reduced the inhibitory effect of FF extract on proliferation (**Supplementary Figure S2F**). Taken together, these results provide preliminary evidence supporting the functional interplay between FF and its key target genes.

### 3.7 FF suppresses HBV-related HCC tumor growth in a xenograft mouse model

**Figure 7:**
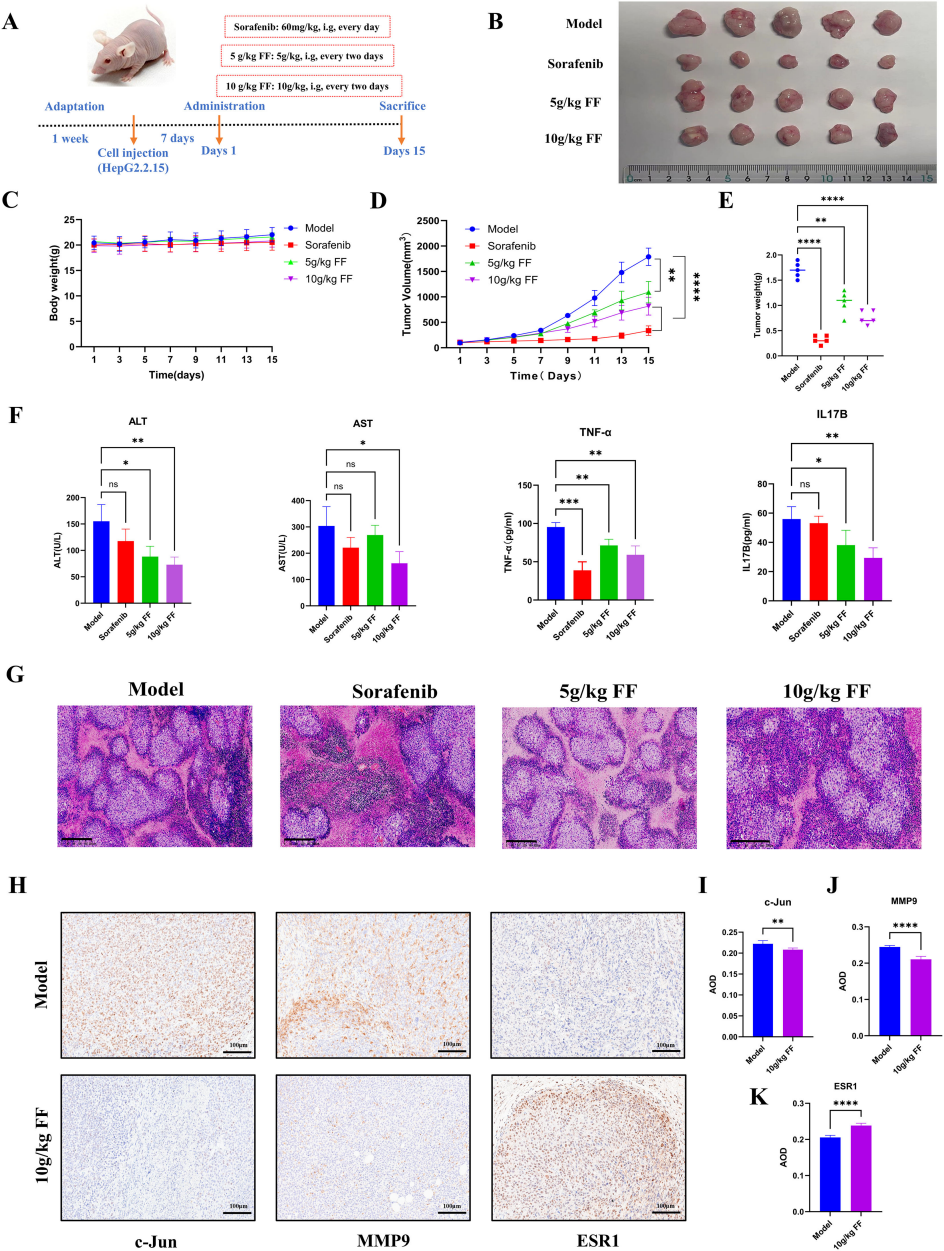
*In vivo* inhibitory effects of FF on HBV-related HCC. **(A)** Schematic representation of the establishment of the mouse model and drug administration for subcutaneous xenograft tumors. **(B)** Physical images of dissected tumors from subcutaneous xenografts in each group (n = 5). **(C)** Changes in body weight of mice in each group during the course of drug administration. **(D)** Tumor growth curves derived from tumor volume measurements. **(E)** Quantitative analysis of tumor weights in each group. **(F)** Serum levels of ALT, AST, TNF-α, and IL-17B in each group. **(G)** Representative microscopic images of H&E-stained tumor tissues in each group (100× magnification; scale bar = 200 μm). **(H)** Representative immunohistochemical images of c-Jun, MMP9, and ESR1 expression in the model and 10 g/kg FF treated groups (200× magnification; scale bar = 100 μm). **(I-K)** The expressions of c-Jun, MMP9 and ESR1 proteins in tumor tissues of the model group and FF group were statistically analyzed. *p < 0.05, **p < 0.01, ***p < 0.001, ****p < 0.0001,ns: no significant difference.

To evaluate the inhibitory effect of FF on tumor growth *in vivo*, we established a xenograft tumor model by subcutaneously injecting HepG2.2.15 cells into the flanks of BALB/c nude mice. The treatment groups comprised normal saline (control), Sorafenib (60 mg/kg, positive control), and FF (5 or 10 g/kg) (**Figure 7A**). Consistent with *in vitro* results, FF treatment significantly reduced tumor volume, size, and weight. Moreover, no significant body weight differences were observed between the model group and FF-treated groups (**Figures 7B–E**). Serum biochemical analysis revealed that FF significantly lowered ALT and AST levels, indicating hepatoprotective effects. Furthermore, FF suppressed serum TNF-α levels, suggesting anti-inflammatory activity. These findings implied that the IL-17 signaling pathway may mediate the therapeutic effects of FF against HBV-related HCC. Thus, we measured serum IL-17B levels to further validate this hypothesis. FF treatment led to a marked downregulation of serum IL-17B (**Figure 7F**). Histopathological examination showed that model group tumors exhibited dense cell packing, abundant cytoplasm, and minimal necrosis. In contrast, Sorafenib- and FF-treated tumors displayed extensive necrosis with abundant cellular debris (**Figure 7G**). IHC demonstrated that high-dose FF (10 g/kg) markedly suppressed c-Jun and MMP9 expression but upregulated ESR1, aligning with *in vitro* data (**Figures 7H–K**). These results collectively suggest that FF exerts anti-HBV-related HCC effects *in vivo*.

## 4 Discussion

The pharmacological effects of FF’ active components collectively contribute to its therapeutic potential against HBV-related HCC. The representative terpenoid β-amyrin acetate protects hepatocytes through its antioxidant mechanisms (49). The lignan phillyrin inhibits viral replication, while the flavonoid quercetin may suppress tumor growth by regulating cell cycle progression (50, 51). These multi-component actions provide a comprehensive phytochemical basis for FF’ traditional use in hepatoprotection and its observed anti-HBV-related-HCC effects in our study. FF has emerged as a promising anti-tumor agent in the TCM, demonstrating broad-spectrum inhibitory effects against various cancer types. In this study, we integrated network pharmacology, molecular docking, MD simulations, combined with *in vitro* and *in vivo* experiments to systematically evaluate the efficacy of FF against HBV-related HCC and elucidate its molecular mechanisms. Our network pharmacology analysis revealed that FF’s therapeutic effects against HBV-related HCC are mediated through 42 potential target genes and 10 major signaling pathways. Using comprehensive network pharmacology approaches, we identified three pivotal genes (c-Jun, ESR1, and MMP9) closely associated with HBV-related HCC pathogenesis and progression, including the highly bioactive compound bicuculline. Moreover, our findings demonstrate the essential role of the IL-17 signaling pathway in FF-mediated anti-HCC effects.

The IL-17 signaling pathway has been increasingly recognized as a key player in HCC progression. Recent study has shown that IL-17RB is overexpressed in clinical HCC samples and its high expression correlates with poor patient prognosis (46).The IL-17 signaling pathway primarily consists of IL-17 family cytokines (such as IL-17A to F) and their corresponding receptors (including IL-17RA to IL-17RE). By binding with these receptors, the IL-17 signaling pathway initiates TRAF6-dependent transcription of target genes and TRAF6-independent mRNA stability mediated by IKKi, thereby activating downstream signaling pathways such as NF-kB, JNK, and MAPK. This activation cascade has been implicated in promoting tumor cell survival, angiogenesis, and immune evasion, which aligns with our observations (52, 53). This intricate cascade plays a pivotal role in host defense, autoimmune diseases, and the pathological mechanisms of cancer development (54). Through KEGG pathway enrichment analysis, we identified significant enrichment of multiple common targets in the IL-17 signaling pathway. Western blot experiments confirmed that FF extract modulates the expression of IL-17RB and the phosphorylation levels of downstream signaling molecules, ERK1/2 and p38 MAPK, in the IL-17 signaling pathway. Furthermore, FF significantly decreased the level of the IL-17B cytokine in mouse serum, suggesting that FF can inhibit the IL-17B-IL17RB signaling axis, thereby blocking the transmission of downstream signals. Previous studies have demonstrated high expression of IL-17RB in HCC and pancreatic cancer cells, as well as clinical tumor samples (46, 48). Patients with elevated IL-17RB expression exhibit poorer prognoses, underscoring its critical role in tumorigenesis and progression. Upon activation by upstream interleukins, IL-17RB facilitates downstream signal transduction, triggering pathways implicated in cancer progression, including NF-κB, MAPK, and STAT3. This mechanism promotes tumor cell survival, angiogenesis, and suppression of anti-tumor immunity, playing a pivotal role in tumorigenesis (52, 53).

The c-Jun molecule, encoded by the c-Jun gene and belonging to the Jun family of transcription factors, is a pivotal component in the activator protein-1 (AP-1) transcription complex. In HCC, c-Jun has been shown to promote chemotherapy resistance by suppressing apoptotic pathways, and its inhibition can sensitize tumor cells to treatment (55). c-Jun plays a crucial role in diverse biological processes encompassing cellular proliferation, differentiation, and apoptosis (54, 56). During early carcinogenesis stages, c-Jun represses c-Fos gene expression, thereby reducing acetyltransferase SIRT6 levels while augmenting survivin expression within cells. Ultimately, this molecular cascade promotes the survival of tumor-initiating cells and drives tumorigenesis. This mechanism is critical in the early stages of various cancers, including HCC (57). In the context of HCC and other malignancies, c-Jun promotes chemotherapy resistance by suppressing cellular apoptosis and other signaling pathways. Targeting c-Jun activity could improve the efficacy of chemotherapy drugs and reverse chemoresistance in cancer treatment (55). The ESR1 gene encodes estrogen receptor alpha (ESR1), a member of NR3 subfamily of nuclear hormone receptors, and regulates target cell proliferation, differentiation, and homeostasis (58). Loss of ESR1 expression has been linked to increased HCC risk, and estrogen signaling has been proposed as a protective mechanism against hepatocarcinogenesis (59, 60). ESR1 expression is frequently downregulated or lost in HCC cells, suggesting a potential protective role of estrogen signaling in hepatocarcinogenesis. The attenuation or loss of ESR1 expression may impair estrogen signaling, thereby contributing to HCC pathogenesis (60). Previous studies have demonstrated that ESR1 exerts hepatoprotective effects during liver carcinogenesis by delaying hepatocyte apoptosis, ameliorating hepatic fibrosis, and inhibiting tumor growth via ESR1-mediated signaling, thereby reducing HCC susceptibility (59). Matrix metalloproteinase-9 (MMP9), a zinc-dependent endopeptidase, is a member of the matrix metalloproteinase family. Elevated MMP9 levels are strongly associated with HCC invasion and metastasis, and its inhibition has been shown to reduce tumor aggressiveness (61). It functions as an enzyme capable of degrading collagen and other components of the extracellular matrix. Extensive research has demonstrated a strong correlation between elevated MMP9 expression and the invasiveness, metastasis, and unfavorable prognosis of HCC (62). Moreover, inhibition of MMP9 activity significantly suppresses the growth and metastatic potential of HCC cells, offering novel insights into HCC treatment strategies (61). In this study, c-Jun, ESR1, and MMP9 were identified as key target genes of FF in the treatment of HBV-related HCC. Significant differences in their expression levels were observed in relation to survival prognosis analysis. Functional verification via gene overexpression elucidated distinct mechanisms underlying the action of FF: ESR1 acts as a tumor suppressor by enhancing FF’s anti-proliferative effect; conversely, c-Jun and MMP9 exhibit pro-cancer characteristics by counteracting FF’s activity. Specifically, c-Jun overexpression promoted cell viability and partially reversed the growth inhibitory effect induced by FF. Although MMP9 had minimal impact on baseline proliferation, it significantly attenuated FF’s efficacy, potentially due to its primary role in promoting tumor invasion and metastasis. These findings underscore a dynamic balance mechanism wherein FF’s therapeutic efficacy depends on the interplay between its core targets and the equilibrium of their opposing functions. Moreover, compared with normal hepatocytes, HepG2.2.15 cells exhibited significant differences in the expression levels of these three core genes. Both *in vitro* and *in vivo* studies demonstrated that FF can effectively target and regulate these genes, providing a molecular basis for the treatment of HBV-related HCC.

In this study, molecular docking analysis revealed that Bicuculline exhibits the highest binding affinity to the target gene compared to the other nine high-quality compounds. Meanwhile, the results of MD simulations further confirmed that the small molecule Bicuculline exhibits excellent binding stability with the target proteins JUN, ESR1, and MMP9. Bicuculline, also known as (+)-Bicuculline or d-Bicuculline, is an alkaloid derived from plants such as Northeastern Stephania. It exhibits significant neuropharmacological properties and acts as a competitive antagonist of the neurotransmitter GABAA receptor (63). By blocking Ca2+-activated potassium (SK) channels, it effectively inhibits slow afterhyperpolarization (slow AHP), demonstrating anticonvulsant activity. This alkaloid holds substantial value in the investigation of various neurological disorders (64). Bicuculline has recently been demonstrated to exhibit potent anti-HCC effects via multi-target mechanisms (65), consistent with our network pharmacology and molecular docking findings. These results further underscore its potential therapeutic value in HCC treatment.

This study also possesses certain limitations and deficiencies. Firstly, network pharmacology primarily relies on data obtained from public databases for analysis. However, these databases may exhibit issues such as incomplete, inaccurate, or outdated information. For instance, the compilation of active ingredients in FF extract might not be sufficiently comprehensive and could potentially overlook some crucial components. Secondly, molecular docking is a theoretical computational approach that can anticipate the binding mode and affinity between drug constituents and target proteins. Nevertheless, these predictions do not necessarily guarantee absolute accuracy. Therefore, subsequent studies need to utilize a series of experimental techniques, such as surface plasmon resonance (SPR) and isothermal titration calorimetry (ITC), to confirm and verify the interactions between these targets and small molecule drugs. Thirdly, the interaction relationships between the three core genes and FF require further validation. In subsequent studies, we will employ siRNA to knock down the expression of these core genes and utilize a multi-dimensional experimental approach to comprehensively elucidate their specific interaction mechanisms and functional associations. Finally, the pharmacological effects of the key active ingredient, Bicuculline, in FF require further validation.

## 5 Conclusion

**Figure 8:**
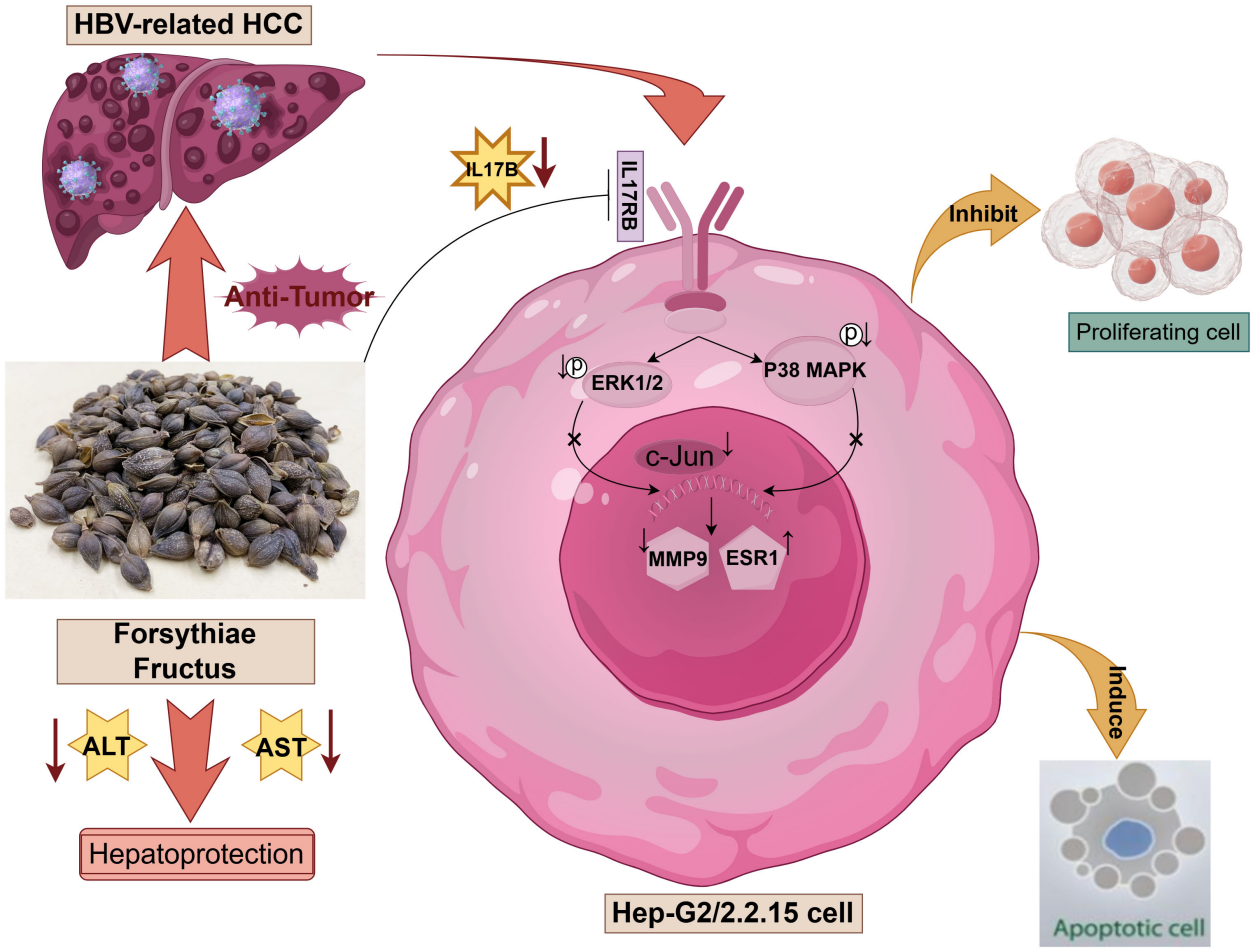
Schematic representation of the molecular mechanism underlying the regulatory effects of Forsythiae Fructus on hepatocellular carcinoma (HCC) related to hepatitis B virus (HBV), created by Figdraw.

In this study, we investigated the potential mechanisms of FF in inhibiting HBV-related HCC through network pharmacology analysis, molecular docking, and *in vitro* and *in vivo* experiments. As illustrated in **Figure 8**, FF may exert its anti-cancer effects by inhibiting the IL-17B-IL17RB signaling axis and concurrently reducing the phosphorylation levels of downstream signaling molecules ERK1/2 and P38 MAPK. Molecular docking, MD simulations, as well as *in vitro* and *in vivo* experimental results have demonstrated that FF can target the three key proteins c-Jun, ESR1, and MMP9. These results offer a more comprehensive insight into the potential mechanisms of action of FF in the treatment of HBV-related HCC.

## Data Availability

The original contributions presented in the study are included in the article/**Supplementary Material**. Further inquiries can be directed to the corresponding author.
