# Supplementary material for: Network pharmacology integrated with molecular docking and experimental validation elucidates the therapeutic potential of Forsythiae Fructus extract against hepatitis B virus-related hepatocellular carcinoma
Source: Front Oncol. 2025 Jun 24;15:1571537. doi: 10.3389/fonc.2025.1571537 (PMC12234312; doi:10.3389/fonc.2025.1571537)
Supplement: Supplementary file 1 [file DataSheet1.docx]

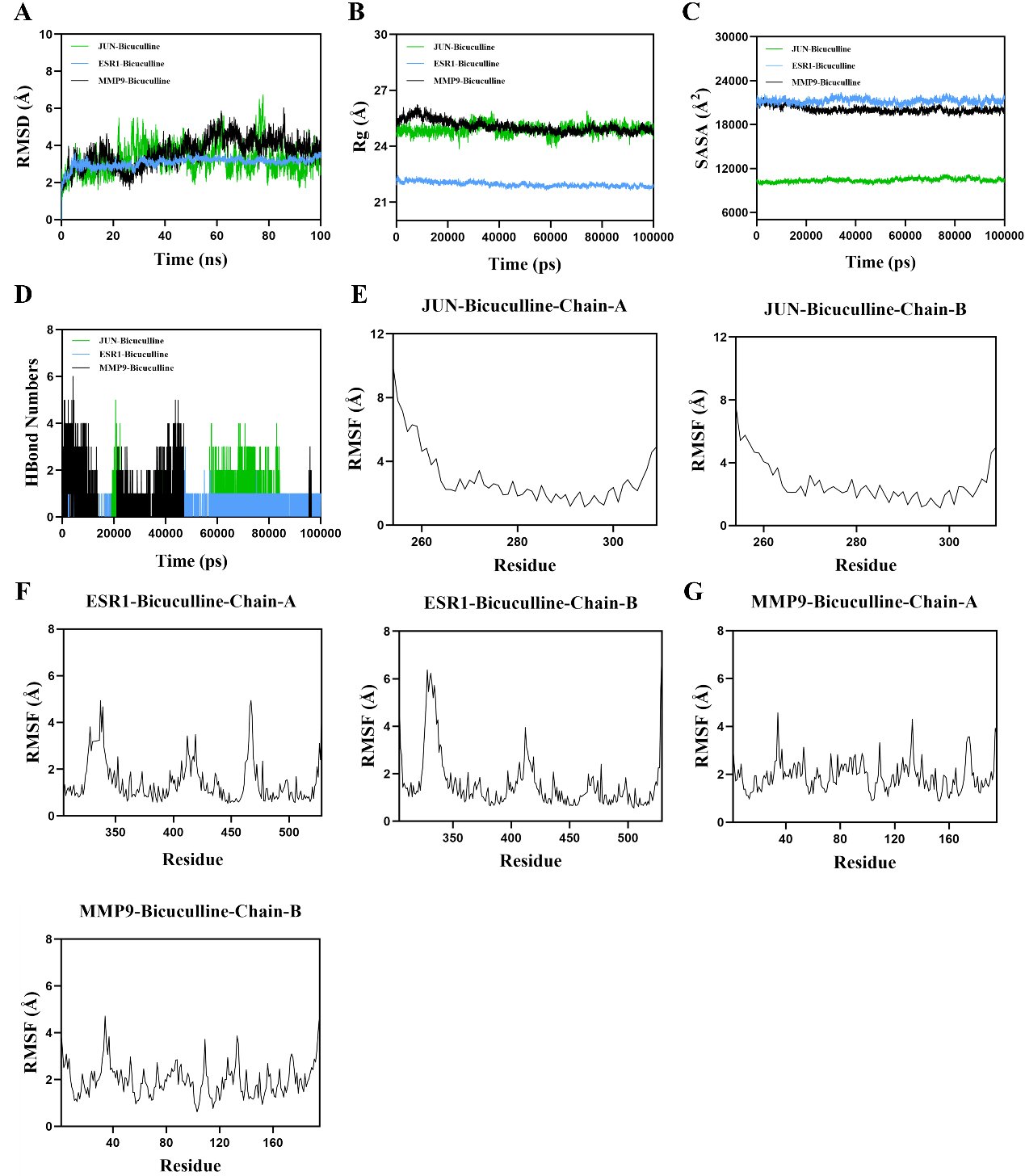


**Figure S1 Molecular dynamics simulation of protein-ligand complex. (A)** Time-dependent RMSD values of the protein-ligand complex. (**B)** Time-dependent Rg values of the protein-ligand complex. (**C)** Time-dependent SASA values of the protein-ligand complex. (**D)** Time-dependent HBonds counts of the protein-ligand complex. (**E)** The changes in the RMSF of the Bicuculline and JUN protein A chain and B chain complexes during the MDS process. (**F)** The changes in the RMSF of the Bicuculline and ESR1 protein A chain and B chain complexes during the MDS process. (**G)** The changes in the RMSF of the Bicuculline and MMP9 protein A chain and B chain complexes during the MDS process. RMSD: root-mean-square deviation; Rg: radius of gyration; SASA: solvent-accessible surface area; HBonds: hydrogen bonds; RMSF: root-mean-square fluctuation. MDS: molecular dynamics simulation.


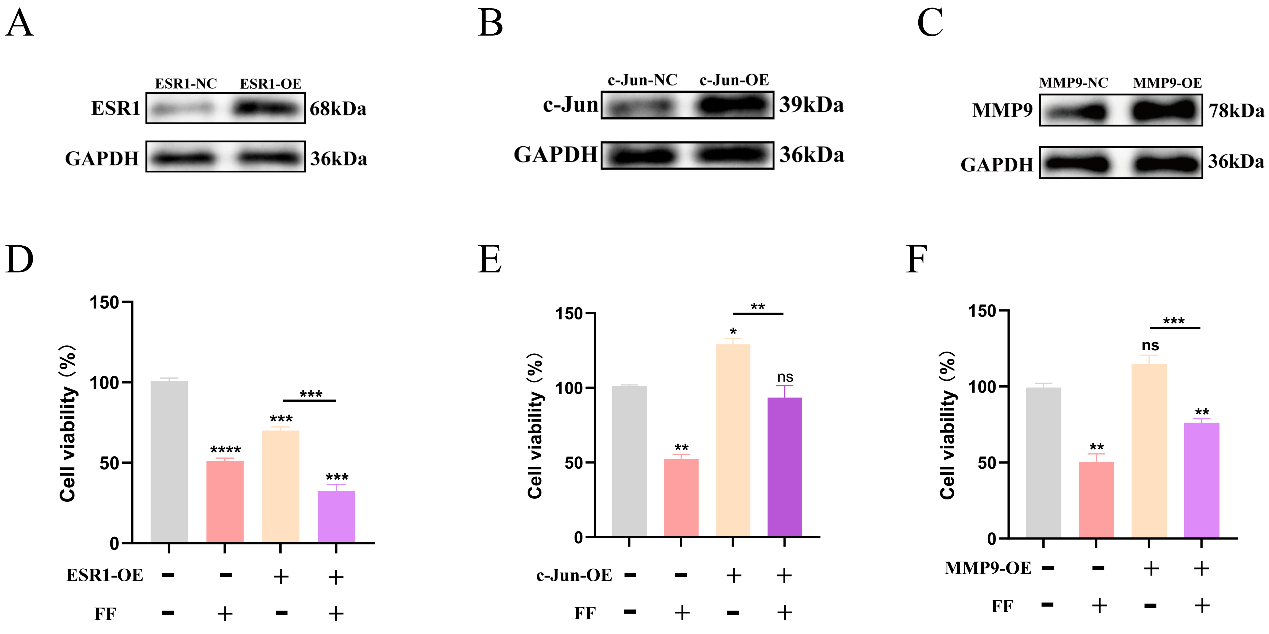


**Figure S2 Analysis of the functional association between FF and core genes.** (**A-C**) The transfection expression levels of ESR1, c-Jun and MMP9 in HepG2.2.15 cells were detected by western blot. (**D**) The effect of FF extract (0.885 mg/mL) on the viability of HepG2.2.15 cells treated with negative control (ESR1 NC) and ESR1 overexpression (ESR1 OE) for 48 hours was investigated. (**E**) The effect of FF extract (0.885 mg/mL) on the viability of HepG2.2.15 cells treated with negative control (c-Jun NC) and c-Jun overexpression (c-Jun OE) for 48 hours was investigated. (**F**) The effect of FF extract (0.885 mg/mL) on the viability of HepG2.2.15 cells treated with negative control (MMP9 NC) and MMP9 overexpression (MMP9 OE) for 48 hours was investigated. FF: *Forsythiae Fructus.* All data are presented as the means ± S.D., *p < 0.05, **p < 0.01, ***p < 0.001, ****p < 0.0001, ns: no significant difference.
